# Light Spectra and Root Stocks Affect Response of Greenhouse Tomatoes to Long Photoperiod of Supplemental Lighting

**DOI:** 10.3390/plants10081674

**Published:** 2021-08-14

**Authors:** Jason Lanoue, Alyssa Thibodeau, Celeste Little, Jingming Zheng, Bernard Grodzinski, Xiuming Hao

**Affiliations:** 1 Harrow Research and Development Centre, Agriculture & Agri-Food Canada, Harrow, ON N0R1G0, Canada; Jason.Lanoue@agr.gc.ca (J.L.); Alyssa.Thibodeau@agr.gc.ca (A.T.); Celeste.Little@agr.gc.ca (C.L.); Jingming.Zheng@agr.gc.ca (J.Z.); 2 Department of Plant Agriculture, University of Guelph, Guelph, ON N1G 2W1, Canada; bgrodzin@uoguelph.ca

**Keywords:** light quality, photoperiod, tomato, light-emitting diode, greenhouse, photosynthesis, light spectra, root stock

## Abstract

Plant biomass and yield are largely dictated by the total amount of light intercepted by the plant (daily light integral (DLI)—intensity × photoperiod). It is more economical to supply the desired DLI with a long photoperiod of low-intensity light because it uses fewer light fixtures, reducing capital costs. Furthermore, heat released by the light fixtures under a long photoperiod extended well into the night helps to meet the heating requirement during the night. However, extending the photoperiod beyond a critical length (>17 h) may be detrimental to production and lead to leaf chlorosis and a reduction in leaf growth and plant vigor in greenhouse tomato production. It is known that red light can increase leaf growth and plant vigor, as can certain rootstocks, which could compensate for the loss in plant vigor and leaf growth from long photoperiods. Therefore, this study investigated the response of tomatoes grafted onto different rootstocks to a long photoperiod of lighting under red and other light spectra. Tomato plants ‘Trovanzo’ grafted onto ‘Emperator’ or ‘Kaiser’ were subjected to two spectral compositions—100% red or a mix of red (75%), blue (20%), and green (5%) light for 17 h or 23 h. The four treatments supplied similar DLI. Leaf chlorosis appeared in all plants under 23 h lighting regardless of spectral compositions between 20 and 54 days into the treatment. The yield for 23 h mixed lighting treatment was lower than both 17 h lighting treatments. However, the 23 h red lighting treatment resulted in less leaf chlorosis and the plants grafted onto ‘Emperator’ produced a similar yield as both 17 h lighting treatments. Therefore, both spectral compositions and rootstocks affected the response of greenhouse tomatoes to long photoperiods of lighting. With red light and proper rootstock, the negative yield impact from long photoperiod lighting can be eliminated.

## 1. Introduction

The daily light integral (DLI; light intensity x photoperiod duration) plays a vital role in plant biomass accumulation and yield. While the natural solar DLI is dictated by time of year, global location, and local weather, the DLI can be augmented by the introduction of supplemental lighting. Supplemental lighting can aid in the achievement of a desired/target DLI to increase plant growth and yield, specifically during low-light months [1]. The use of an extended photoperiod with supplemental light at a lower light intensity can have economic benefits by reducing the overall fixture need (i.e., capital cost) and by using electricity during the night, when electrical costs are low [2]. Furthermore, most of the input electricity in light fixtures is eventually converted into heat because plants only convert a small percentage of light into biomass. By utilizing LEDs during the subjective night period, the heat released from light fixtures can help to meet nighttime heating requirements. However, exceeding the tolerable limits of photoperiods, which are species-specific, can lead to diminished yield, photoperiod-related leaf injury, and an economic disadvantage for growers [3]. For tomatoes, photoperiods up to 17 h are associated with normal growth patterns [4]. Photoperiods beyond 17 h, which do not employ a drastic temperature dip, spectral change, or decrease in light intensity, have been shown to cause photoperiod-related injury characterized by leaf chlorosis, photosynthetic inhibition, and yield decrease [5,6,7]. However, prolonged photoperiods (>18 h) can theoretically lead to increased plant biomass and yield due to the added light available for photosynthesis, if photoperiod-related injury is not induced [8].

The underlying mechanism involved in photoperiod-related injury has yet to be determined. The *type III light harvesting chlorophyll a/b binding protein 13* (*CAB-13*) gene has been demonstrated to play an important role in photoperiod-related injury [9]. As the name suggests, *CAB-13* plays an important role in the light harvesting/photosystem II (PSII) super complex [10]. When plants were grown under continuous light (CL, 24 h), *CAB-13* expression was downregulated, leading to photoinhibition and photoperiod-related injury characterized by a decrease in the maximum quantum efficiency of PSII (F_v_/F_m_) [9]. Velez-Ramirez et al. [9] also suggest that photoperiod-related injury may be due to the unbalanced excitation between photosystem I (PSI) and PSII. Furthermore, Haque et al. [6] hypothesized that continuous lighting could cause damage to PSII, which ultimately reduces photosynthesis and yield. It has also been hypothesized that the restoration of proper leaf photochemistry, potentially through improved expression of genes such as *CAB-13*, as well as balancing source/sink strength may alleviate injury under extended photoperiods [6,9,11,12]. From this, we have deduced that the utilization of different spectral compositions during an extended photoperiod may play a key role in avoiding or lessening photoperiod-related leaf injury.

The ability to regulate gene expression can largely be traced back to the role of photoreceptors such as phytochrome and cryptochrome [13]. With the advancements in light-emitting diode (LED) technology, the interaction between photoperiod length and spectral composition has become of interest in optimizing growth conditions for high-value crops. With red and blue LEDs being the most efficient and these wavelengths being primarily absorbed by phytochrome and cryptochrome, respectively, much research relating to photoperiods and spectra has focused around these wavelengths [7,9,14]. Matsuda et al. [14] indicated that photoperiod-related injury was less severe when tomato seedlings were exposed to red or orange LEDs compared to blue or white during the subjective night period when grown under CL. Moreover, Velez-Ramirez et al. [15] determined that phytochrome A (PHY A) plays an important role in photoperiod-related injury. Together, these studies indicate that light spectral compositions/quality may play an important role in reducing tomato injury under extended photoperiods. However, both Matsuda et al. [14] and Velez-Ramirez et al. [15] performed experiments using controlled environment growth chambers, which would exclude any effect that the natural solar radiation would have. Furthermore, using such chambers would not facilitate adequate growth space for plants to reach maturity (both studies only used young plants up to the first flower stage) and thus did not allow for the assessment of yield during prolonged photoperiods.

Exposing tomatoes to extended photoperiods tends to lead to smaller leaf area [9,16]. It has been stated that even if tomatoes were genetically altered to be CL-tolerant, the overall leaf area would be low, resulting in reduced light capture and plant growth [9]. However, leaf expansion can be controlled by spectral compositions. Red supplemental light is generally thought of as a vegetative light, able to improve leaf expansion, whereas blue light is known to reduce leaf size and increase leaf thickness [17,18]. Therefore, utilizing red light during extended photoperiods may overcome the reduction in leaf size invoked by CL. It should also be noted that the rootstocks can affect plant growth. While grafting is traditionally done to invoke disease resistance, many studies have shown that proper selection of rootstock materials can increase leaf area as well as plant vigor, leading to improved yield [19,20,21]. Therefore, the use of wavelength-specific lighting such as red light and proper rootstock selection may alleviate photoperiod-related injury. For these reasons, we set out to test the response of large vining tomatoes to an extended photoperiod of lighting with different spectral compositions/quality and rootstocks to see if the negative impact of extended photoperiod lighting on tomato fruit production can be eliminated by proper selection of light spectral compositions and rootstocks.

## 2. Materials and Methods

### 2.1. Plant Material and Experimental Design

Tomato (*Solanum lycopersicum*) seedlings cv. ‘Trovanzo’ were grafted onto ‘Emperator’ (TE) or onto ‘Kaiser’ (TK) in a double-stemmed (twin-head) system. ‘Emperator’ has been observed to promote vegetative growth (or a vigorous rootstock) whereas ‘Kaiser’ has been shown to promote generative growth. In this way, we could observe the homeostatic balance between two different rootstock types. Twin-head transplants (5 weeks old) raised by a commercial propagator were placed into rockwool slabs on top of a raised growing trough (30 cm high) in a large glass greenhouse (200 m^2^ growing area) at the Harrow Research and Development Centre, Agriculture and Agri-Food Canada, Harrow, Ontario, Canada (42.03° N, 82.9° W) on 11 November 2018 at a plant density of 2 plants m^−2^ (4 stems m^−2^). The plants were planted into 6 rows, with the 2 outside rows serving as guard rows. The two stems/heads of each plant in the row were trained upward along the vertical strings into a ‘V’ system as in commercial production. The strings were hung onto the top wires (3.5 m high). Once the plant head reached the overhead wires, a few bottom leaves were removed and the plants were lowered twice every week. The plants were drip-irrigated using a complete nutrient solution [22]. The electrical conductivity and pH were set at 2.8 dS m^−1^ and 5.8, respectively. Plants were grown at an enriched CO_2_ concentration of 800 µL L^−1^ when the greenhouse was not ventilated. The average daytime temperature was held between 21 and 24 °C during the months of November, December, and January. Average daytime temperatures during the months of February and March were between 21 and 25 °C. Average daytime temperatures during the months of April and May were between 22 and 27 °C depending on the ambient solar radiation. Nighttime temperature was maintained at 20 ± 1 °C throughout the production period. Relative humidity of 70 ± 10% was maintained during both daytime and nighttime periods.

**Figure 1:**
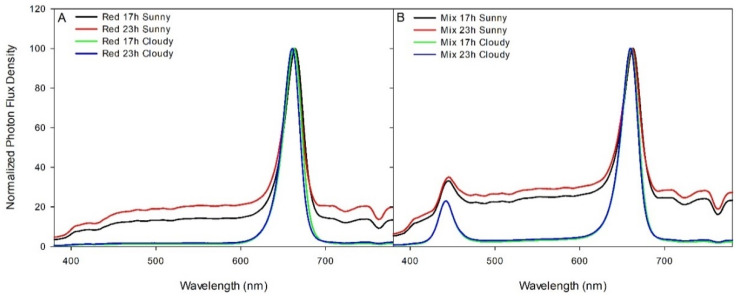
Normalized photon flux density (380–780 nm) from red and mixed LED lighting treatments during both sunny and cloudy days. The spectra were measured using a Li-180 Spectrometer (Li-COR Inc., Lincoln, NE, USA) at a distance of 80 cm from the light fixtures. Measurements for sunny days were done on 11 February 2019 and cloudy days on 14 February 2019 between 12:00 and 14:00. Panel (**A**) represents spectra from both red light treatments during sunny and cloudy days. Panel (**B**) represents spectra from both mixed light treatments during sunny and cloudy days.

The 4 middle rows were divided into 16 plots via white curtains, which were impenetrable to light. There were 24 twin-head plants (48 stems) in each plot; 12 plants for each of the 2 rootstocks were planted. Four supplemental overhead lighting treatments were applied to the 16 plots in a Latin square design with 4 replications (one lighting treatment in each row or column of the 16 plots): 100% red from 23:00 to 16:00 (Red 17 h), 100% red from 17:00 to 16:00 (Red 23 h), mixed light from 23:00 to 16:00 (Mix 17 h), and mixed light from 17:00 to 16:00 (Red 23 h; Figure 1). The mixed light had 75% red (600–700 nm), 20% blue (400–499 nm), and 5% green light (500–599 nm), with green light being introduced via white diodes. The four lighting treatments provided similar DLIs (Table 1). All the lighting treatments were applied using Pro 325e smart LED fixtures from LumiGrow (Emeryville, California, USA). A 23 h monochromatic red photoperiod was utilized because red light tends to preferentially support vegetative growth. Furthermore, red diodes are highly efficient, and thus a pure red spectrum is able to achieve the highest photosynthetic photon efficacy (PPE) [23]. The mixed light spectrum was chosen as a control, which provides close to the recommended amounts of blue light [24] and some green light, which has also been shown to improve tomato growth and yield [25].

Application of the supplemental lighting treatments began on 16 November 2018. Supplemental light intensities as shown in Table 1 were determined at three different positions within a treatment at 80 cm from the light fixtures (just above the heads of the plants) using a Li-COR 190R (Li-COR Biosciences Inc., Lincoln, NE, USA) quantum line sensor during the nighttime period to exclude natural solar radiation. Red:far-red (R:Fr) were determined on both sunny and cloudy days (11 February and 14 February 2019, respectively) by dividing the total photons of red by the total photons of far-red (700–780 nm). Further to this, the phytochrome photostationary state (PSS) was determined using Equation (1) from Sager et al. [26], where *N* is the photon flux (mol m^−2^ s^−1^) and *σ_r_* and *σ_Fr_* are the photochemical cross-section of phytochrome in the red absorbing state and far-red absorbing state, respectively.

(1) $$PSS=\frac{\left(\sum_{380}^{780}N\sigma_{r}\right)}{\left(\sum_{380}^{780}N\sigma_{r}+\sum_{380}^{780}N\sigma_{Fr}\right)}$$

**Figure 2:**
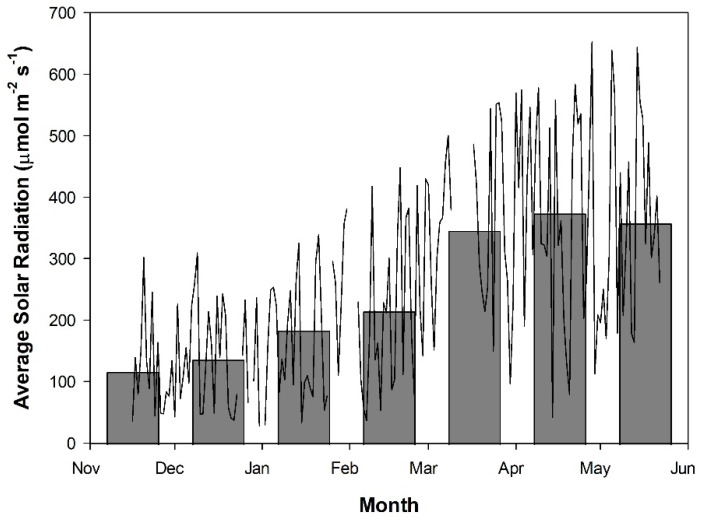
Daily average solar radiation as measured from 16 November 2018 to 22 May 2019 using a Li-COR LI-200R pyranometer converted from W m^−2^ to µmol m^−2^ s^−1^ using the conversion value of 2.1. Readings were taken above the greenhouse and then corrected for an approximate 50% transmissivity to account for shading from the greenhouse structure, lighting fixtures, and shade curtains. Measurements were taken every 2 h, beginning at 08:00 and concluding at 16:00, between the wavelengths of 400 and 1100 nm. Measurements during this period were averaged to provide an average solar radiation for each day (line plot). The bar graph indicates the average daily solar radiation throughout the month. Breaks in the line plot indicate periods of time which were not documented due to a technical malfunction.

Spectral readings for these calculations were taken between 12:00 and 14:00 using a Li-COR Li-180 spectrometer on both sunny and cloudy days under their respective lighting treatments. The sunny day was one with no observable clouds in the sky and the cloudy day was fully overcast. Throughout the experiment, supplemental lighting remained on regardless of ambient light levels to ensure that all treatments received similar total DLIs (Figure 2). The curtains were closed during cloudy days to prevent light contamination between treatments. During sunny days, the curtains were opened to prevent shading of natural light. On days which were partly cloudy/sunny, the forecast was used to determine the majority (i.e., sun or cloudy) and then curtains were opened or closed as appropriate. Because there was sunlight between 16:00 and 17:00 (i.e., during natural sunset), the actual photoperiods (including sunlight) were 18 h and 24 h (CL) for the 17 h and 23 h lighting, respectively. In commercial greenhouses, bumble bees are used as pollinators for fruit setting. The stopping of supplemental lighting between 16:00 and 17:00 was to facilitate the return of bees to their hives under natural dusk/sunset conditions; otherwise, the bees may get lost and significantly increase the number of bees (and associated cost) needed for pollination [27]. Dusk/sunset varied throughout the course of the experiment from 16:52 to 20:22, which was sufficient to allow for bees to return to their hives.

### 2.2. Growth Measurements

Growth measurements were performed on 6 randomly selected plants from TE and TK at 31, 60, and 139 days into the treatment (DIT), corresponding with 16 December 2018, 14 January 2019, and 3 April 2019, respectively. Growth measurements included leaf length, leaf width, and chlorophyll content of the 5th, 10th, and 15th leaf when applicable. Leaf chlorophyll was measured using a SPAD meter (model 502, Konica Minota, Osaka, Japan) and values were converted to chlorophyll content using correction equations generated by spectrophotometric pigment analysis. Chlorophyll correction curves were generated by extracting leaf punches in 95% ethanol at 78 °C for approximately 3 h until the tissue was cleared. Samples were then analyzed at 664.2 nm, 648.6 nm, and 470 nm wavelengths using a spectrophotometer (Beckman DU-640 UV–Vis, Indianapolis, IN, USA). Concentrations of *chlorophyll a*, *b*, and carotenoids were determined via equations from [28].

### 2.3. Leaf Gas Exchange: Day and Night Measurements

The 5th leaves from TE plants were placed in the chamber of a Li-COR 6400 (Li-COR Inc., Lincoln, NE, USA), which was fitted with a 2 cm × 3 cm clear-top chamber. The leaf temperature was set to 24 °C, with a relative humidity of 55–65% and a CO_2_ level held at 800 µL L^−1^. Three leaves from separate plants under each treatment were used at 21 DIT (6 December 2018) and 55 DIT (9 January 2019) for both daytime and nighttime measurements. Measurements were taken during the day on cloudy days to maximize the effect of supplemental lighting while minimizing the effect of natural light. Nighttime measurements were taken between 18:00 and 20:00, which was at least 1 h after sunset on each respective day. This was done to ensure that plants had sufficient time to reach a steady-state photosynthetic or respiratory rate depending on treatment. Leaves were kept in the chamber until steady-state photosynthesis rates were obtained; then, the average from a 2-min period was taken.

### 2.4. Leaf Gas Exchange: Light Response Curves

The 5th leaves from TE plants were placed in the chamber of a Li-COR 6400, which was fitted with a 2 cm × 3 cm red/blue LED Li-COR standard light source (88Red/12%Blue). The leaf temperature was set to 24 °C, with a relative humidity of 55–65% and a CO_2_ level held at 800 µL L^−1^. Three leaves from separate plants under each treatment were used at 20, 54, and 134 DIT, corresponding with 5 December 2018, 8 January 2019, and 29 March 2019, respectively. Measurements were performed on cloudy days. Light curves began at a high light intensity and decreased gradually following the procedure from Lanoue et al. [29]. At each light level, the photosynthetic rate was allowed to reach a steady state; then, a measurement was taken for that light level. Photosynthetic rates were plotted against light intensity and fitted to a regression line following the equation y = y_o_ + a(1−e^(−b*x)^), using SigmaPlot 10.0 to determine the photosynthetic maximum. A linear regression (y = mx + b) using the photosynthetic rates at the light levels of 0–100 µmol m^−2^ s^−1^ was used to calculate both the light compensation point (LCP) and quantum yield (QY).

### 2.5. Leaf Gas Exchange: CO_2_ Response Curves

The 5th leaves from TE plants were placed in the chamber of a Li-COR 6400, which was fitted with a 2 cm × 3 cm red/blue LED Li-COR standard light source (88%R/12%B). The leaf temperature was set to 24 °C, with a relative humidity of 55–65% and a light level of 300 µmol m^−2^ s^−1^. CO_2_ response curves were specifically preformed at a light level of 300 µmol m^−2^ s^−1^ to assess the leaf photosynthetic capacity at, or near, an average growth condition. Thus, the maximum rate of Rubisco carboxylation (V_cmax_) and the maximum rate of electron transport (J_max_) are associated with a non-saturated light level. Three leaves from separate plants under each treatment were used at 20, 54, and 134 DIT, corresponding with 5 December 2018, 8 January 2019, and 29 March 2019, respectively. Measurements were performed on cloudy days. CO_2_ response curves began at the ambient CO_2_ concentration (800 µL L^−1^) and reduced gradually to 50 µL L^−1^. After the 50 µL L^−1^ measurement, the CO_2_ concentration was set to 800 µL L^−1^ and was held steady until plant photosynthetic parameters returned to levels established during the beginning of the experiment. The CO_2_ level was then increased incrementally to 2000 µL L^−1^, at which point the CO_2_ response curve was terminated. At each CO_2_ concentration, the photosynthetic rate was allowed to reach a steady state; then, a measurement was taken to produce values for that CO_2_ concentration. Photosynthetic rates were plotted against internal CO_2_ concentration (C_i_) and fitted to the FvCB model [30] and temperature-corrected [31,32] to determine the maximum rate of photosynthesis under Rubisco-limited and RuBP-limited conditions.

### 2.6. Chlorophyll Fluorescence Imaging

Intact leaflets from TE plants were dark-adapted using aluminum foil for 10 min. After the dark adaptation period, leaflets were detached and immediately used for chlorophyll imaging using a closed FluorCam model FC 800-C with FluorCam v.7.0 software (FluorCam, Photon System Instruments, Brno, Czech Republic). The minimum fluorescence in a dark-adapted state (F_o_) was acquired during a dark period of 5 s, after which an 800 ms saturating light pulse (2400 µmol m^−2^ s^−1^) from a blue LED (peak emission of 449 nm) was used to measure maximum fluorescence in a dark-adapted state (F_m_). From F_o_ and F_m_, the variable fluorescence in a dark-adapted state (F_v_) was calculated (F_v_ = F_m_ − F_o_), which was used to determine the maximum photosystem II (PSII) quantum yield (F_v_/F_m_). In general, the lower the value of F_v_/F_m_ is, the more severe the photoinhibition and thus the leaf injury [33]. By calculating F_v_/F_m_ using chlorophyll fluorescence imaging, we were able to assess not only the prevalence of injury but also the spatial heterogeneity of F_v_/F_m_ from a leaflet. Eight leaflets from the 5th leaf were used for each lighting treatment when plants were 23, 62, and 138 DIT, corresponding with 8 December 2018, 16 January 2019, and 2 April 2019, respectively.

### 2.7. Fruit Yield

First harvest began on 1 February 2019, 78 DIT. Clusters of tomato were harvested when 4 out of the 5 fruits in the cluster had become red, twice a week. The harvested fruit was graded according to commercial grading standards [22]. The fruit number and weight for marketable and unmarketable fruit were recorded from 1 February 2019 to 17 May 2019. On 22 May 2019, the plants were strip-harvested. During the strip harvest, all tomatoes regardless of ripeness stage were harvested and used during calculations. The plants were topped (growing head removed) 4 weeks before the strip harvest. By the time of strip harvest, all the clusters of fruit on the plants had reached full size but had still not reached commercial harvest stage yet (80% red). Throughout the manuscript, yield data from 1 February 2019 to 28 February 2019 are designated as February; yield data from 1 March 2019 to 31 March 2019 are designated March; yield data from 1 April 2019 to 30 April 2019 are designated April; and yield data from 1 May 2019 to the strip harvest on 22 May 2019 are designated May.

### 2.8. Statistical Analysis

All statistics were performed using SAS Studio 3.5. Means comparisons between the red 17 h, red 23 h, mix 17 h, and mix 23 h lighting treatments were done using a two-way ANOVA assessing the effects of photoperiod length, spectral quality, and the interaction with a Tukey–Kramer adjustment and a *p* < 0.05 indicating a significant difference.

## 3. Results

### 3.1. Chlorophyll Fluorescence and Photosynthesis

**Figure 3:**
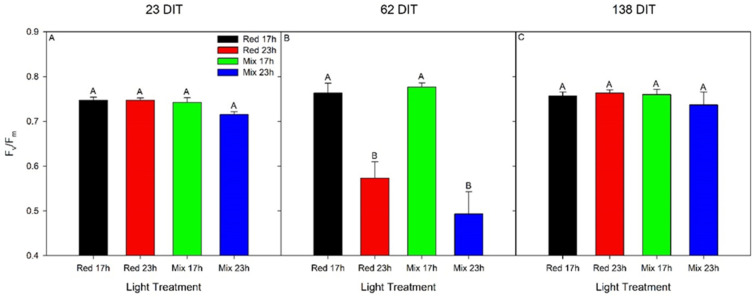
Maximum efficiency of PSII (F_v_/F_m_) from the 5th leaf of TE tomatoes grown under red 17 h, red 23 h, mix 17 h, or mix 23 h lighting treatments at 23 DIT (8 December 2018, panel **A**), 62 DIT (January 16, panel **B**), and 138 DIT (2 April 2019, panel **C**). Error bars represent the standard error of the mean of n = 8. Letter groups (A, B) represent significant differences between the lighting treatments at a specific time point and leaf position at *p* < 0.05.

**Figure 4:**
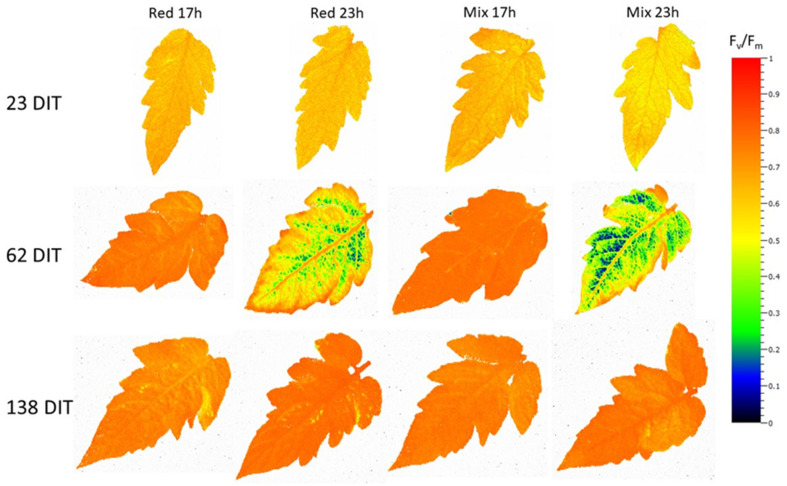
Spatial response of F_v_/F_m_ from the 5th leaf of TE tomatoes grown under either red 17 h, red 23 h, mix 17 h, or mix 23 h lighting treatments at 23 DIT (8 December 2018), 62 DIT (16 January 2019), and 138 DIT (2 April 2019).

Assessing the maximum efficiency of PSII (F_v_/F_m_) via chlorophyll fluorescence measurements is often used as a proxy measurement to assess the health of a plant. Here, it was used to assess injury related to photoperiod extension. During the initial stage of growth (23 DIT, 8 December 2018), leaves from TE tomato plants had similar maximum efficiency of PSII (Figure 3A). At 62 DIT (16 January 2019), leaves exposed to both red 23 h and mix 23 h lighting treatments produced lower F_v_/F_m_ values compared to leaves exposed to red 17 h and mix 17 h treatments (Figure 3B). During the late stage of growth (138 DIT, 2 April 2019), leaves under all lighting treatments again produced similar F_v_/F_m_ values (Figure 3C). Of note, leaves under both red 23 h and mix 23 h treatments showed an increase in F_v_/F_m_ values from 62 DIT to 138 DIT to values similar to those at the beginning of the experiment, indicating full recovery. As shown in Figure 4, leaves exposed to red 23 h and mix 23 h lighting displayed photoinhibition patterns characteristic of interveinal chlorosis. These patterns were not apparent on the 5th leaf of any treatments at 138 DIT (Figure 4). Leaves under the mix 23 h lighting treatment tended to have more interveinal chlorosis than leaves under the red 23 h lighting treatment at 62 DIT (Figure 4), which is also seen by a slightly higher F_v_/F_m_ in Figure 3B.

**Figure 5:**
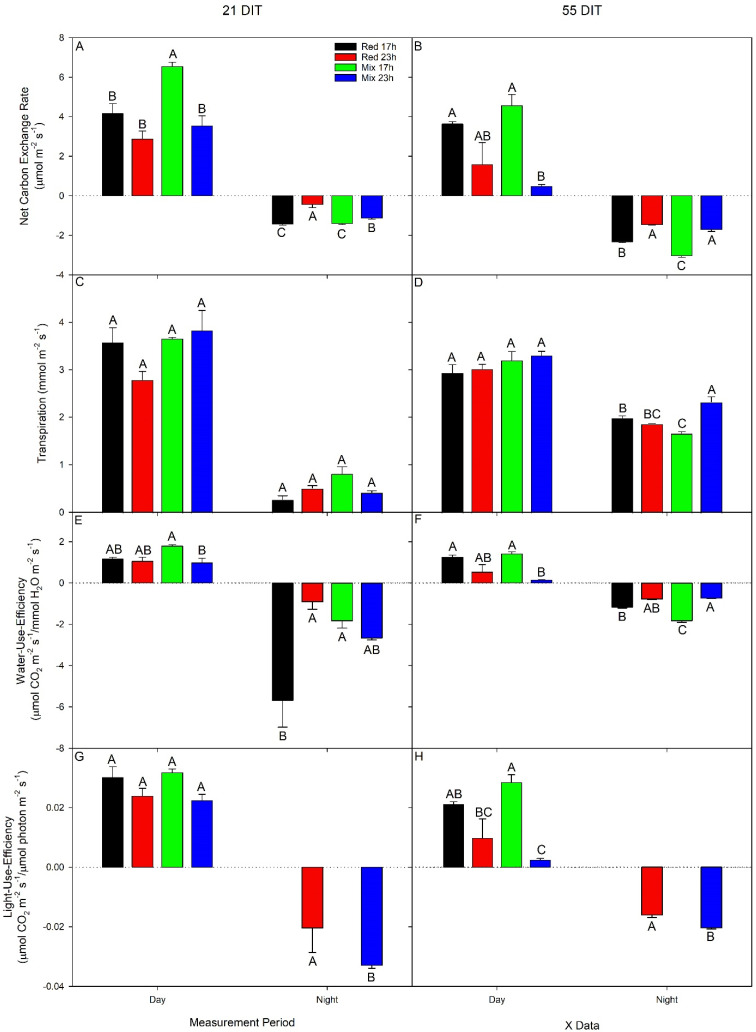
Net carbon exchange rate (NCER; panel **A**,**B**), transpiration (panel **C,D**), water use efficiency (panel **E**,**F**), light use efficiency (panel **G**,**H**) of the 5th leaf from TE tomato plants grown under either red 17 h, red 23 h, mix 17 h, or mix 23 h lighting treatments at 21 DIT (6 December 2018, panels **A**,**C**,**E**,**G**) or 55 DIT (9 January 2019, panels **B**,**D**,**F**,**H**) during the daytime and nighttime. Measurements were performed using a Li-COR 6400 fitted with a clear-top chamber on a cloudy day or night and thus represent the NCER driven mostly by the supplemental lighting. Error bars represent the standard error of the mean of n = 3. Letter groups (A, B, C) represent significant differences within a panel between the lighting treatments at a specific data collection period at *p* < 0.05.

At 21 DIT (6 December 2018), leaves under the mix 17 h treatment produced the highest daytime net carbon exchange rate (NCER; Figure 5A). Both red 23 h and mix 23 h as well as the red 17 h treatment produced statistically lower daytime NCER values compared to leaves exposed to mix 17 h. Respiration rates, indicated by a negative NCER, were the highest under both 17 h lighting treatments (Figure 5A). In both 23 h lighting treatments, an increase in NCER was observed as there was light from the LEDs during this subjective nighttime period (Figure 5A). It should be noted that the red 23 h lighting treatment had a higher NCER than the mix 23 h lighting treatment during the subjective nighttime period, which may indicate the first signs of the alleviation of photoperiod-related injury by red light (Figure 5A). At 55 DIT (9 January 2019), leaves exposed to mix 23 h produced drastically reduced daytime NCER compared to leaves exposed to mix 17 h lighting treatment (Figure 5B), indicating severe damage caused by the long photoperiod with a mixed light spectrum. However, leaves exposed to red 23 h produced statistically similar daytime NCER values as both 17 h lighting treatments. Leaves exposed to both 17 h lighting treatments produced the highest respiration rates during the nighttime period, as expected (Figure 5B). Surprisingly, leaves under both 23 h lighting treatments also produced negative NCER values (indicating respiration), even during a period with an appreciable amount of supplemental light (Figure 5B). However, it should be noted that the respiratory rate in both 23 h treatments was lower than those in the 17 h treatment. While light was present in the night, it might not have been utilized to the full extent due to the leaf chlorosis (Figure 3B and Figure 4).

Daytime transpiration rates at both 21 DIT and 55 DIT were similar among all lighting treatments (Figure 5C,D). At 21 DIT, nighttime transpiration rates were also similar among all lighting treatments (Figure 5C). However, at 55 DIT, the transpiration rate was the highest in leaves exposed to the mix 23 h lighting treatment and the lowest under the mix 17 h treatment (Figure 5D). Water use efficiency (WUE) indicates the rate of CO_2_ and H_2_O exchange through stomata, with a positive rate indicating photosynthesis and a negative rate indicating respiration. At 21 DIT and 55 DIT, the daytime WUE of leaves exposed to the mix 17 h lighting treatment was higher than leaves exposed to the mix 23 h lighting treatment (Figure 5E,F). At 55 DIT, WUE from leaves exposed to the red 17 h treatment was also higher than leaves under the mix 23 h treatment. However, WUE was not different between 17 h and 23 h with red light. Therefore, light spectral compositions did affect the response of WUE to the long photoperiod (23 h). Nighttime WUE at 21 DIT was the lowest under the red 17 h treatment and the highest under both red 23 h and mix 17 h (Figure 5E). At 55 DIT, nighttime WUE was the lowest in leaves exposed to the mix 17 h lighting treatment but the highest under the mix 23 h lighting treatment (Figure 5F). 

Light use efficiency (LUE) is the calculation of how much CO_2_ is fixed per incoming unit of photons. In this way, it provides a metric which allows for the assessment of light capture and carbon fixation. All leaves at 21 DIT produced similar LUE values (Figure 5G). At 55 DIT, leaves grown under both 17 h lighting treatments produced higher LUE values than leaves under the mix 23 h lighting treatment (Figure 5H). At both time periods, nighttime LUE for the 17 h treatments was non-resultant due to a light intensity of 0 µmol m^−2^ s^−1^ (Figure 5G,H). At 21 DIT, the nighttime LUE was higher under the red 23 h treatment than the mix 23 h lighting treatments (Figure 5G). Similar results were obtained during measurements at 55 DIT (Figure 5H). This indicates that leaves grown under an extended red photoperiod were better able to utilize the light during the subjective nighttime period than those under the mixed spectrum, likely due to less photoperiod-related injury.

**Figure 6:**
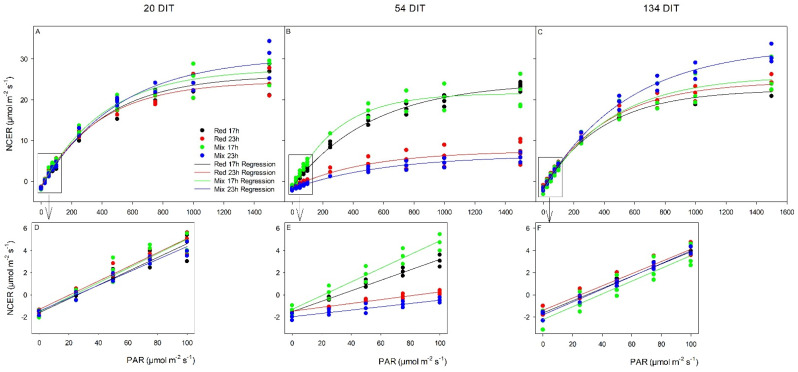
Photosynthetic light response curves from TE leaves grown under either red 17 h, red 23 h, mix 17 h, or mix 23 h lighting treatments at 20 DIT (5 December 2018, panel **A**), 54 DIT (8 January 2019, panel **B**), and 134 DIT (29 March 2019, panel **C**) as determined using a Li-COR 6400 with a red/blue standard Li-COR light source. Measurements were performed at a CO_2_ concentration of 800µL L^−1^, leaf temperature of 24 °C, and a relative humidity of 55–65%. Regression lines were fit to y = y_o_ + a(1 − e^(−b*x)^) for each lighting treatment. Panels (**D**–**F**) are magnifications of 0–100 µmol m^−2^ s^−1^ PAR regions fit to the regression line y = mx + b.

At 20 DIT (5 December 2018), photosynthetic light response curves were generated for the fifth leaf of TE tomatoes (Figure 6). All photosynthetic parameters (i.e., respiration rate, light compensation point (LCP), quantum yield (QY), and maximum photosynthetic rate (Pn_max_)) were similar among all treatments (Table 2). At 54 DIT (8 January 2019), leaves exposed to the mix 17 h lighting treatment produced the lowest respiration rate and leaves exposed to the mix 23 h lighting treatment produced the highest (Table 2). Both 23 h lighting treatments produced drastically higher LCP than leaves exposed to the 17 h treatments, showing an inability to utilize light well (Figure 6F; Table 2). At 54 DIT, leaves exposed to the mix 17 h treatment produced the highest QY out of all lighting treatments. Furthermore, leaves exposed to the red 17 h treatment produced higher QY than leaves exposed to either red 23 h or mix 23 h treatments (Table 2). At 54 DIT, Pn_max_ was greatly reduced in both 23 h lighting treatments compared to both red 17 h and mix 17 h lighting treatments (Table 2). At 134 DIT (29 March 2019), respiration rate, LCP, and QY were similar between all lighting treatments. However, Pn_max_ was higher in leaves exposed to the mix 23 h lighting treatment than the red 17 h lighting treatment (Table 2).

**Figure 7:**
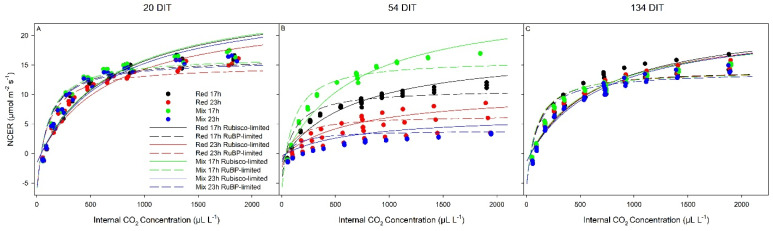
Photosynthetic CO_2_ response curve from leaves grown under red 17 h, red 23 h, mix 17 h, and mix 23 h lighting treatments at 20 DIT (5 December 2018, panel **A**), 54 DIT (8 January 2019, panel **B**), and 134 DIT (29 March 2019, panel **C**). As determined using a Li-COR 6400 with a red/blue standard Li-COR light source. Measurements were performed at 300 µmol m^−2^ s^−1^ PAR, a temperature of 24 °C, and relative humidity of 55–65%. Rubisco- and RuBP-limited fit lines were determined using temperature corrections from [31,32].

At 20 DIT (5 December 2018), all parameters related to photosynthetic performance (V_cmax_ and J_max_) were similar among all lighting treatments (Figure 7; Table 3). At 54 DIT (8 January 2019), leaves exposed to the mix 17 h lighting treatment produced the highest V_cmax_, J_max_, and Pn_max_ compared to the other lighting treatments (Table 3). During the same time period, leaves exposed to the red 17 h lighting treatment produced higher values of V_cmax_, J_max_, and Pn_max_ than leaves exposed to either 23 h lighting treatment (Table 3). At 134 DIT (29 March 2019), V_cmax_, J_max_, and Pn_max_ were similar among all lighting treatments, returning to levels observed at the beginning of the experiment (Table 3).

### 3.2. Plant Parameters

Leaf length and width were measured in TE and TK plants at 31 DIT (16 December 2018) and resulted in similar values between two rootstocks for both metrics (Table 4). At 60 DIT (14 January 2019), the 5th and the 10th leaf from TE produced similar lengths and widths, respectively, under each lighting treatment (Table 4). However, the fifth leaves from TK exposed to the red 17 h lighting treatment were longer than those same leaves exposed to the mix 23 h treatment (Table 4), indicating that the rootstock can affect the response to the lighting treatments. This was not observed with leaf width, where all treatments produced the same width for the fifth leaves of TK plants. The interaction between lighting treatments and rootstocks demonstrates a competitive advantage during the use of extended photoperiods. This highlights the importance of selecting lighting treatments and rootstocks which combat the leaf area reduction typically observed under CL to improve light capture. Both the leaf length and leaf width of the 10th leaf at 60 DIT were similar in leaves exposed to all lighting treatments of TK plants (Table 4). At 139 DIT (3 April 2019), similar leaf lengths and widths were observed under all lighting treatments for both TE and TK (Table 4).

At 31 DIT (16 December 2018), leaves from both rootstocks produced similar total chlorophyll and carotenoid concentrations when exposed to all lighting treatments (Table 5). At 60 DIT (14 January 2019), TE leaves grown under the mix 17 h lighting treatment produced the highest total chlorophyll and carotenoid concentrations of any lighting treatments at both the 5th and 10th leaf position (Table 5). TK leaves grown under either the red 17 h or mix 17 h lighting treatments produced higher total chlorophyll concentrations than leaves grown under the mix 23 h lighting treatment at the fifth leaf position (Table 5), indicating that mix 23 h caused leaf chlorophyll reduction when grafted onto ‘Kaiser’ (TK). At the 10th leaf position, leaves grown under the mix 17 h lighting treatment produced a higher total chlorophyll concentration than did leaves grown under the mix 23 h lighting treatment at the same position (Table 5). At the fifth leaf position of TK leaves at 60 DIT, those grown under the mix 23 h lighting treatment produced the lowest carotenoid concentration of any lighting treatment (Table 5). At 139 DIT (3 April 2019), leaves from TE plants produced similar total chlorophyll and carotenoid concentrations at each leaf position under each lighting treatment (Table 5). Leaves at the fifth leaf position from TK plants had higher total chlorophyll and carotenoid concentrations when grown under the mix 17 h treatment than either the red 17 h or red 23 h lighting treatment at 139 DIT (Table 5). Overall, plants grown under the mix 17 h lighting treatment produced high levels of pigments related to light capture and photosynthesis.

### 3.3. Fruit Yield

**Figure 8:**
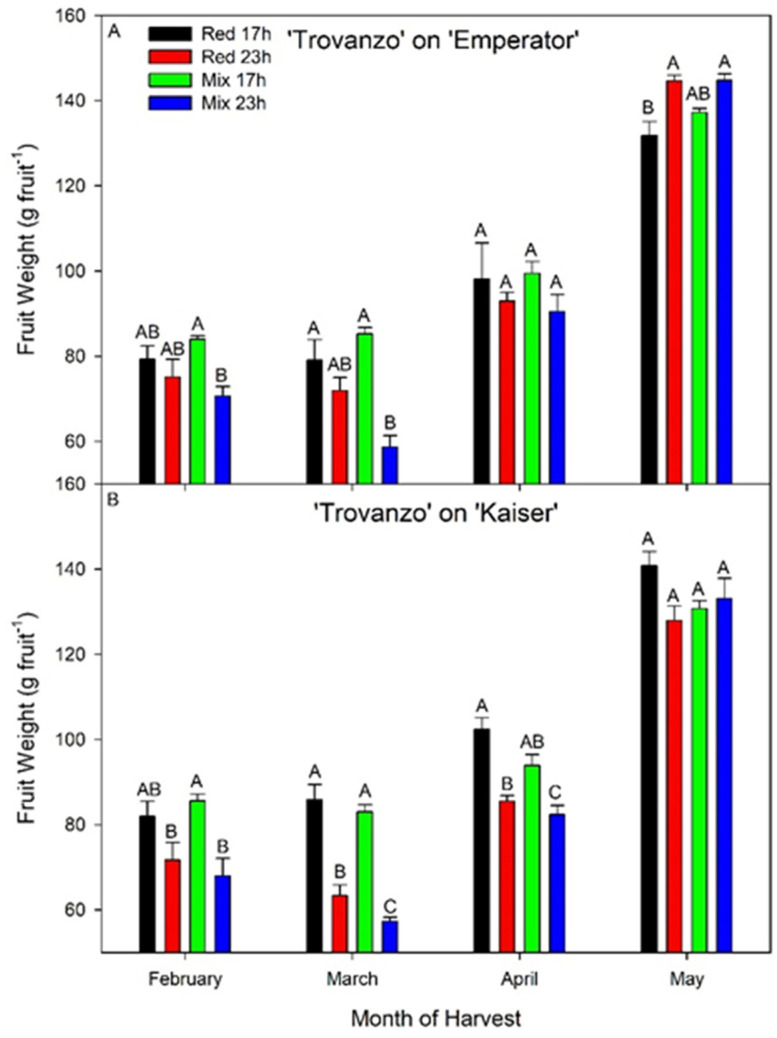
Average fruit weight (size) for cv. ‘Trovanzo’ grafted onto ‘Emperator’ (TE; panel **A**) or onto ‘Kaiser’ (TK; panel **B**), grown under red 17 h, red 23 h, mix 17 h, and mix 23 h lighting treatments. Monthly fruit number includes the 1st of each month to the last day of each respective month. Values represent the mean ± the standard error of the mean, where n = 4 for TE and TK. Of note, values representing May yield include a strip harvest on 22 May 2019. Within the month and cultivar, letter groups (A, B, C) represent a statistical difference in fruit weight as determined by a two-way ANOVA with a Tukey–Kramer adjustment (*p* < 0.05).

During the month of February, the average fruit weight (size, g fruit^−1^) from TE plants was similar between red 17 h, red 23 h, and mix 17 h (Figure 8A). Notably, plants grown under the mix 23 h treatment produced a lower fruit weight than plants under the mix 17 h treatment during February (*p* = 0.0046; Figure 8A). During the month of March, the average fruit weights for TE plants grown under the red 17 h and mix 17 h lighting treatments were observed to be higher than plants grown under the mix 23 h lighting treatment (*p* = 0.0022; Figure 8A). In April, TE plants produced a similar average fruit weight among all lighting treatments (Figure 8A). During May, plants grown under both red 23 h and mix 23 h supplemental lighting treatments produced a higher average fruit weight than plants grown under the red 17 h treatment (*p* = 0.0022; Figure 8A).

In February, TK plants under the mix 17 h lighting treatment produced a higher average fruit weight than did plants grown under either red 23 h and mix 23 h treatments (*p* = 0.003; Figure 8B). During the month of March, plants grown under the red 17 h and mix 17 h lighting treatments produced similar average fruit weight (Figure 8B). Plants under both 17 h lighting treatments produced higher average fruit weights than did the 23 h lighting treatments (*p* < 0.0001; Figure 8B). Notably, plants grown under the mix 23 h lighting treatment produced the lowest fruit weight of any treatments (Figure 8B). During the month of April, plants grown under the red 17 h treatment produced higher average fruit weight than plants grown under the red 23 h lighting treatment (*p* = 0.0036; Figure 8B). Furthermore, plants grown under both 17 h lighting treatments produced higher fruit weights than did plants under the mix 23 h lighting treatment (*p* = 0.0025; Figure 8B). In May, the average fruit weight was similar among all lighting treatments (Figure 8B).

Throughout the harvest period (i.e., from 1 February 2019 to 22 May 2019), within a rootstock and lighting treatment, fruit production increased each month (Table 6) as more sunlight became available. In the month of February, within each rootstock, all lighting treatments produced similar values of fruit number per stem (Table 6). TE plants grown under both 23 h lighting treatments produced the lowest total fruit weight per stem during the month of February (*p* = 0.0091; Table 6). During the month of March, TE plants produced the same number of fruits per stem under all lighting treatments (Table 6). However, TK plants grown under both 23 h lighting treatments produced low numbers of fruits per stem, with plants grown under the mix 23 h treatment being the lowest of all treatments (*p* = 0.0006; Table 6), indicating that 23 h lighting caused more damage with TK than TE. TE plants grown under the mix 17 h lighting treatment produced higher fruit weight per stem than plants grown under the mix 23 h lighting treatment (*p* = 0.0018; Table 6). TK plants grown under both 17 h lighting treatments had higher fruit weight per stem than both 23 h lighting treatments, with plants grown under the mix 23 h lighting treatment having the lowest overall (*p* < 0.0001; Table 6). Importantly, during the month of March, both TE and TK plants grown under the mix 23 h lighting treatment had the lowest total fruit weight per stem (Table 6).

During the month of April, both TE plants (*p* = 0.0019) and TK plants (*p* = 0.0003) grown under the mix 23 h lighting treatment produced the lowest total fruit weight per stem (Table 6). However, total fruit weight per stem from TE under the red 23 h was similar to both 17 h lighting treatments while the total fruit weight per stem from TK under the red 23 h lighting treatment was still lower than the 17 h red lighting treatment. The above results indicate that both light spectra and rootstocks played a role in photoperiod-related injury as an improved yield recovery time was observed from TE compared to TK. During the month of May, both TE and TK plants produced similar values of both total fruit number per stem and fruit weight per stem, indicating the full recovery of fruit production under the 23 h lighting treatments (Table 6).

For both TE (*p* = 0.0029) and TK (*p* = 0.0001) plants, growth under the mix 23 h lighting treatment was associated with the lowest cumulative number of fruit per stem throughout the entire production period (Table 6). Similarly, for TE (*p* = 0.0017) and TK (*p* < 0.0001), plants grown under the mix 23 h lighting treatment produced the lowest cumulative fruit weight per stem (Table 6). It should be noted that TE plants grown under the red 23 h lighting treatment produced similarly high cumulative fruit number and weight per stem to both 17 h treatments, whereas TK plants under the red 23 h still produced a lower weight per stem than the 17 h treatments (Table 6). This again shows that TK plants tended to be more affected by the extended photoperiod than TE plants, indicating an effect of rootstock material on photoperiod-related injury. Taken together, these results suggest that the use of a broad (mix) spectrum lighting treatment during an extended photoperiod tends to have a more negative effect on fruit yield than a monochromatic red spectrum.

## 4. Discussion

### 4.1. Effect of Photoperiod Extension on Leaf Physiology

Extending the natural solar photoperiod via the implementation of supplemental lighting has proven to be a beneficial lighting strategy in terms of plant growth and yield during greenhouse production of tomatoes [1]. However, decades of research have shown that photoperiod extension beyond a critical length causes photoperiod-related injury characterized by leaf chlorosis [9,34,35,36]. Indeed, this negative result observed under photoperiods greater than 17 h has nullified any theoretical advantages they may have [8].

The increased use of wavelength-specific LED fixtures during controlled environment plant production has the potential to play a pivotal role in reducing injury related to photoperiod extension. During CL, Matsuda et al. [14] indicated that young tomato plants (23 days after planting and 13 days into the treatment) grown under white light during the day and either red or orange light during the subjective nighttime period produced a lower degree of injury than plants grown under white light during the day and either white or blue light at night. A recent study by Lanoue et al. [7] produced injury-free tomatoes during a 6-month production period grown under CL by using an alternating spectrum of red during the day and blue during the subjective nighttime period. However, a reduction in the light level also occurred during the spectral shift from 200 µmol m^−2^ s^−1^ of red light to 50 µmol m^−2^ s^−1^ of blue light (close to light compensation point), which may have confounded any effects of the spectral shift [9]. In this study, the light intensity was maintained at the same level for the two lighting treatments, allowing for the comparison of spectral compositions/quality without any confounding effects.

During the initial stages of plant growth, plants grown under all lighting treatments were observed to be injury-free (Figure 3A and Figure 4). However, as determined by measurements between 54 and 62 DIT (8–16 January 2019), plants grown under either red 23 h or mix 23 h treatments developed photoperiod-related injury, characterized by leaf chlorosis, consistent with previous research (Figure 3B and Figure 4) [6,35,36,37]. At this period (around 2–4 weeks before the starting of fruit harvest—78 DIT; it usually takes 2 weeks for a cluster of fruit from mature green (full size) to reach harvesting stage—4 red fruits out of the 5 fruits in each cluster), the clusters of fruits were in the fastest growing stage (fastest increase in size). The plants had heavy fruit sink and weak leaf source due to low natural sunlight (Figure 2, 8–16 January 2019). During this period of measurements, parameters related to photosynthesis, such as LCP, QY, Pn_max_, V_cmax_, and J_max_, were all reduced from leaves that were exposed to both 23 h lighting treatments compared to those exposed to either 17 h lighting treatment (Table 2 and Table 3). The downregulation in parameters related to photosynthesis may be related to either excess carbohydrate accumulation or a downregulation of *CAB-13*, affecting PSII function [9,38]. Interestingly, during this period of production, which was characterized by photoperiod-related injury and a downregulation in parameters related to photosynthesis, both stomatal conductance and transpiration rates were unaffected (Figure 5). It should be noted that Lanoue et al. [7] did not observe issues with carbon metabolism during CL. Therefore, the major driver of injury during extended photoperiods cannot simply be attributed to improper stomatal function or overaccumulation of carbohydrates. Furthermore, as a decrease in the chlorophyll content was observed (Table 5), photoperiod-related injury seems to be more closely related to light capture.

From 54 DIT (8 January 2019) onwards, plants and leaves grown under both 23 h treatments were observed to recover from the photoperiod-related injury (Figure 3C and Figure 6C). The photosynthetic capacity and F_v_/F_m_ values from leaves under 23 h treatments returned to levels similar to the 17 h treatments, and fruit yield during the months of April and May also increased (Table 6). However, no changes to the experimental settings had occurred to invoke such changes. What did change was the peak intensity of natural solar radiation (Figure 2) and the natural photoperiod from January to April. In fact, from the period of peak photoperiod injury (62 DIT, 16 January 2019) to when photoperiod injury was completely alleviated (138 DIT, 2 April 2019), the solar radiation nearly doubled in average daily intensity from 88 W m^−2^ to 165 W m^−2^ (Figure 2). Moreover, during this period, the plant growth was more balanced—less fruit load due to fruit harvesting and more leaf growth due to strong sunlight. The changes in both solar radiation and plant growth balance (generative vs. vegetative or source vs. sink) may, in some way, account for the observed recovery of plants under both 23 h lighting treatments. Further specifically designed studies will be needed to separate the light intensity effect from that of fruit load/plant growth stage. Potentially, as the natural light intensity increased later in the study, the strong exogenous signaling (i.e., high peak light intensity) was able to override the endogenous signal causing injury [34,37,39]. However, an in-depth look at this hypothesis was beyond the scope of our study.

### 4.2. Effect of Photoperiod Extension on Fruit Production

Photoperiod-related leaf injury characterized by chlorosis and a downregulation of photosynthesis can drastically reduce fruit yield due to a decrease in carbon assimilation essential for plant growth. However, if photoperiod-related injury can be avoided, an increase in plant growth and yield is theoretically possible [8]. The use of wavelength-specific LEDs has shown promise in reducing photoperiod-related injury during the vegetative growth stage [14]; however, little is known about how the use of wavelength-specific LEDs will affect the yield of high-wire-fruiting vegetables during extended photoperiods [7,40]. Between the months of February and April, those which followed the photoperiod-related injury in leaves, the average fruit weight (size, g fruit^−1^) was observed to decrease in both 23 h lighting treatments (Figure 5 and Figure 8). These data indicate a clear correlation between the onset and persistence of injury and the reduction in fruit production. Notably, by April, all yield parameters for both 23 h lighting treatments were similar to those of the 17 h lighting treatments (Figure 8 and Table 6), again coinciding with increased natural solar radiation.

Throughout the harvest period in both TE and TK, those under the red 23 h lighting treatment tended to show less yield reduction than plants under the mix 23 h treatment when compared to the 17 h lighting treatments (Figure 8 and Table 6). In fact, our cumulative yield analysis indicates that growth under the red 23 h lighting treatment of both TE and TK plants produced higher yield than did plants grown under the mix 23 h lighting treatment (Table 6). Furthermore, TE and TK plants grown under the mix 23 h lighting treatment produced fewer fruits per stem than all other lighting treatments (Table 6). These results indicate that the use of supplemental red light during photoperiod extension may be more beneficial than a mixed lighting treatment including appreciable amounts of blue light (Figure 1).

Generally, under a traditional 16 h photoperiod, the addition of some blue light (6–12%) to a predominantly red supplemental lighting spectrum has shown increases in biomass and total fruit number in greenhouse tomatoes [24]. For this reason, in commercial production practices, the addition of blue light is typically thought of as generative light, while increasing the red light component is thought to promote vegetative growth (i.e., vegetative light). In this way, growers can steer the plant towards a more generative or vegetative growing pattern depending on current/future environmental and plant conditions. Administering light during an extended, nearly continuous photoperiod can also be described as a generative light environment as there is constant photon energy pressuring the plant during the subjective nighttime period—in effect, forcing growth. Thus, we hypothesize that the interaction of a mixed spectrum with an extended photoperiod may cause imbalances in plant growth patterns, while using a pure red, more vegetative spectrum during an extended photoperiod may allow for proper vegetative vs. generative homeostasis. We believe that because of the use of the more vegetative red 23 h lighting treatment, the plants grown under this treatment displayed better yield performance than did the more generative mix 23 h treatment (Figure 8 and Table 6). Therefore, the interaction between photoperiod and light spectrum needs to be taken into account during tomato production.

### 4.3. Interaction between Rootstocks and Photoperiod Length

While wild tomato species are generally tolerant to extended photoperiods, domesticated tomatoes have been determined to be sensitive to extended photoperiods (i.e., photoperiod-related leaf injury has been observed) [9]. Our study involved the use of one domesticated tomato cultivar as the scion (‘Trovanzo’) and two domesticated tomato cultivars as rootstocks (‘Emperator’ and ‘Kasier’). Interestingly, the interaction between light spectra and photoperiod impacted the severity of injury—and, ultimately, yield—differently between rootstocks. The TE (‘Trovanzo’ grafted on ‘Emperator’) plants were observed to be more tolerant to photoperiod extension than the TK (‘Trovanzo’ grafted onto ‘Kaiser’) plants (Figure 8 and Table 6). It should also be noted that TE plants had statistically similar yield under the red 23 h lighting treatment as both 17 h lighting treatments; this was not the case for TK plants (Figure 8 and Table 6).

Rootstock material can have a large impact on the growing patterns of the plant as a whole [19,20,21]. Rahmatian et al. [20] showed that overall plant biomass and yield could be increased simply by grafting the same scion onto different rootstock materials, demonstrating the impact that proper rootstock selection can have. In muskmelon and orange trees, the use of different rootstocks also led to differences in fruit production and carbohydrate status in the fruit [41,42]. These studies indicate that the source vs. sink balance can also be impacted by the rootstock selection [20,21,41,42]. This then can have an impact on photoperiod-related injury due to the improper balance between overall vegetative and generative plant growth. In our study, we used two different rootstocks that have previously demonstrated generative (‘Kasier’) and vegetative (‘Emperator’) growing patterns. TE plants generally performed better in terms of fruit yield during extended photoperiods than TK plants, indicating that rootstocks were interacting with the light environment in some fashion. Velez-Ramirez et al. [43] showed that when a CL-sensitive scion was grafted onto a CL-tolerant rootstock, the CL-sensitive scion was less affected by CL. However, in Velez-Ramirez et al.’s study [43], the CL-tolerant rootstock was also allowed to grow a shoot along with the CL-sensitive scion. This allowed the CL-tolerant accession to have leaves exposed to the CL and thus the authors proposed that a “transferable injurious substance” or a signaling molecule could be transferred between the CL-tolerant and CL-sensitive accession [43]. In our study, no vegetative shoots from the rootstock were allowed to grow; thus, the rootstocks were not able to interact with the light environment directly. In a recent study, Paponov et al. [44] observed the modulation of phytohormones in the root zone due to varied canopy light environments. Thus, during growth under extended photoperiods, it is important to take into account the rootstocks being used and not simply the scions as there may be potential communication via hormone signaling involved in the regulation of photoperiod-related injury [45].

## 5. Conclusions

In conclusion, our data indicate that tomatoes grown under extended photoperiods under sole red supplemental light have improved fruit yield compared to those grown under a mixed lighting treatment. In fact, growth under a 23 h photoperiod with red supplemental light resulted in similar cumulative yield parameters to plants grown under a red or mixed light spectrum with 17 h lighting. By utilizing a low intensity and long photoperiod with energy-efficient red LEDs, a decrease in capital fixture cost and electrical costs can be realized. Under both red and mixed 23 h lighting treatments, physiological parameters indicated the presence of photoperiod-related injury at 54 DIT (8 January 2019) during the measurement period. However, during the subsequent production period, photoperiod-related injury was alleviated. Because no experimental parameters were altered, we propose that the increase in peak solar intensity or overall DLI from January to April could play a role in alleviating injury, but further study will be needed to confirm this hypothesis. Interestingly, the rootstocks used were observed to have an interaction with the light spectra and photoperiod length, which led to improved yield from TE plants compared to TK plants. These data suggest that both rootstocks and light spectral compositions/quality can impact photoperiod-related injury. With red light and proper rootstock, the negative yield impact from long photoperiod lighting can be eliminated.

## Figures and Tables

**Table 1:** Photosynthetic photon flux density of supplemental lighting treatments (400–700 nm; 80 cm below the LED fixtures) as determined by using a Li-190R quantum line sensor at night. Red to far-red ratios (R:Fr) were estimated by dividing the total photons of red (600–700 nm) by the total photons of far-red (700–780 nm) during both sunny and cloudy days when the supplementary lighting fixtures were on. Phytochrome photostationary state (PSS) was calculated using Equation (1) [26].

Lighting Treatment	Supplemental Light Intensity (µmol m^−2^ s^−1^)	R:Fr—Sunny Day	R:Fr—Cloudy Day	PSS—Sunny Day	PSS—Cloudy Day	Supplemental DLI (mol m^−2^ d^−1^)
Red 17 h	176 ± 7	3.78	25.56	0.82	0.88	10.79 ± 0.41
Red 23 h	127 ± 4	2.81	17.77	0.80	0.87	10.54 ± 0.36
Mix 17 h	169 ± 4	2.55	15.29	0.79	0.86	10.32 ± 0.27
Mix 23 h	134 ± 5	2.30	12.01	0.78	0.86	11.09 ± 0.42

**Table 2:** Summary of the major physiological traits as determined by leaf light response curves (Figure 6) from tomatoes grown under red 17 h, red 23 h, mix 17 h, and mix 23 h at 20 (5 December 2018), 54 (8 January 2019), and 134 DIT (29 March 2019). Respiration values were the averages of NCER when the light level was 0 µmol m^−2^ s^−1^. The light compensation point (LCP) and quantum yield (QY) were calculated from a regression line (y = mx + b) fitted to the values between the PAR values of 0–100 µmol m^−2^ s^−1^. The photosynthetic maximum (Pn_max_) was calculated from y = y_o_ + a(1 − e^(−b*x)^). Values ± the standard error of the mean are representative of n = 3. Within each parameter and time of measurement, letter groups (A, B, C, D) represent a statistical difference as determined by a two-way ANOVA with a Tukey–Kramer adjustment (*p* < 0.05).

Lighting Treatment	Respiration (µmol CO_2_ m^−2^ s^−1^)	LCP (µmol m^−2^ s^−1^)	QY (µmol CO_2_ m^−2^ s^−1^/ µmol m^−2^ s^−1^)	Pn_max_ (µmol CO_2_ m^−2^ s^−1^)
**20 DIT**				
**Red 17 h**	−1.61 ± 0.24 ^A^	26.23 ± 3.71 ^A^	0.062 ± 0.006 ^A^	28.34 ± 2.13 ^A^
**Red 23 h**	−1.31 ± 0.13 ^A^	20.79 ± 2.33 ^A^	0.064 ± 0.005 ^A^	25.97 ± 2.67 ^A^
**Mix 17 h**	−1.52 ± 0.38 ^A^	23.07 ± 5.64 ^A^	0.065 ± 0.005 ^A^	29.59 ± 2.16 ^A^
**Mix 23 h**	−1.47 ± 0.22 ^A^	25.12 ± 3.08 ^A^	0.058 ± 0.004 ^A^	29.68 ± 1.23 ^A^
**54 DIT**				
**Red 17 h**	−1.65 ± 0.05 ^B,C,D^	32.48 ± 2.59 ^B^	0.047 ± 0.003 ^B^	25.80 ± 0.23 ^A^
**Red 23 h**	−1.45 ± 0.04 ^A,B,C^	86.14 ± 7.37 ^A^	0.017 ± 0.002 ^C^	8.88 ± 1.88 ^B^
**Mix 17 h**	−1.38 ± 0.24 ^A,B^	21.45 ± 5.13 ^B^	0.062 ± 0.002 ^A^	23.87 ± 2.76 ^A^
**Mix 23 h**	−1.98 ± 0.18 ^C,D^	131.60 ± 10.21 ^A^	0.015 ± 0.0003 ^C^	8.24 ± 1.10 ^B^
**134 DIT**				
**Red 17 h**	−1.71 ± 0.08 ^A^	29.58 ± 0.93 ^A^	0.055 ± 0.002 ^A^	24.19 ± 1.24 ^B^
**Red 23 h**	−1.45 ± 0.24 ^A^	25.44 ± 4.78 ^A^	0.055 ± 0.002 ^A^	25.93 ± 1.03 ^AB^
**Mix 17 h**	−2.31 ± 0.49 ^A^	39.00 ± 9.12 ^A^	0.058 ± 0.002 ^A^	28.15 ± 2.36 ^AB^
**Mix 23 h**	−1.83 ± 0.24 ^A^	31.08 ± 3.30 ^A^	0.058 ± 0.002 ^A^	35.00 ± 1.36 ^A^

**Table 3:** Summary of the major physiological traits as determined by leaf CO_2_ response curves (Figure 7) from tomatoes grown under red 17 h, red 23 h, mix 17 h, and mix 23 h at 20 (5 December 2018), 54 (8 January 2019), and 134 DIT (29 March 2019). The maximum rate of Rubisco carboxylation (V_cmax_) and the maximum rate of electron transport (J_max_) were determined using equations from [31,32]. Pn_max_ was calculated from y = y_o_ + a(1−e^(−b*x)^) and indicates the maximum rate of photosynthesis at a light level of 300 µmol m^−2^ s^−1^ at a saturating CO_2_ level. Values ± the standard error of the mean are representative of n = 3. Within each parameter and time of measurement, letter groups (A, B, C) represent a statistical difference as determined by a two-way ANOVA with a Tukey–Kramer adjustment (*p* < 0.05).

Lighting Treatment	V_cmax_ (µmol CO_2_ m^−2^ s^−1^)	J_max_ (µmol e^−^ m^−2^ s^−1^)	Pn_max_ (µmol CO_2_ m^−2^ s^−1^)
**20 DIT**			
**Red 17 h**	26.98 ± 1.24 ^A^	100.16 ± 8.26 ^A^	19.71 ± 0.82 ^A^
**Red 23 h**	24.48 ± 0.66 ^A^	85.61 ± 4.22 ^A^	18.26 ± 0.53 ^A^
**Mix 17 h**	27.19 ± 0.45 ^A^	103.18 ± 3.04 ^A^	20.13 ± 0.31 ^A^
**Mix 23 h**	26.23 ± 0.29 ^A^	95.80 ± 1.64 ^A^	19.35 ± 0.20 ^A^
**54 DIT**			
**Red 17 h**	17.71 ± 0.46 ^B^	55.61 ± 1.67 ^B^	13.07 ± 0.31 ^B^
**Red 23 h**	10.56 ± 2.11 ^C^	30.84 ± 6.66 ^C^	7.80 ± 1.52 ^C^
**Mix 17 h**	26.05 ± 0.20 ^A^	96.30 ± 1.58 ^A^	19.08 ± 0.14 ^A^
**Mix 23 h**	6.54 ± 0.14 ^C^	18.26 ± 0.39 ^C^	4.97 ± 0.12 ^C^
**134 DIT**			
**Red 17 h**	23.17 ± 1.38 ^A^	80.98 ± 7.66 ^A^	17.00 ± 1.03 ^A^
**Red 23 h**	22.33 ± 0.51 ^A^	79.51 ± 2.48 ^A^	17.02 ± 0.39 ^A^
**Mix 17 h**	21.76 ± 0.10 ^A^	78.50 ± 0.64 ^A^	16.72 ± 0.11 ^A^
**Mix 23 h**	22.77 ± 0.90 ^A^	77.00 ± 3.36 ^A^	16.69 ± 0.53 ^A^

**Table 4:** Leaf parameters of plants grown under red 17 h, red 23 h, mix 17 h, and mix 23 h at 31 (16 December 2018), 60 (14 January 2019), and 139 DIT (3 April 2019). Values ± the standard error of the mean are representative of n = 6 for TE and TK plants under all lighting treatments. TE values are under white columns while TK values are under shaded columns. Different letter groups (A, B) represent a statistical difference between rootstocks and lighting treatments within a time point, leaf rank, and leaf parameter at *p* < 0.05.

		Leaf Length (cm)		Leaf Width (cm)	
Cultivar		TE	TK	TE	TK
**Lighting Treatment**	Leaf Rank				
**31 DIT**					
**Red 17 h**	5th	46.33 ± 0.80 ^A^	47.83 ± 1.25 ^A^	36.00 ± 1.51 ^A^	42.50 ± 2.17 ^A^
**Red 23 h**	5th	46.17 ± 1.14 ^A^	43.83 ± 1.89 ^A^	39.83 ± 0.98 ^A^	35.67 ± 3.69 ^A^
**Mix 17 h**	5th	45.83 ± 0.95 ^A^	47.67 ± 1.69 ^A^	45.83 ± 0.95 ^A^	38.67 ± 1.74 ^A^
**Mix 23 h**	5th	42.67 ± 1.43 ^A^	44.50 ± 1.18 ^A^	42.67 ± 1.43 ^A^	38.67 ± 2.08 ^A^
**60 DIT**					
**Red 17 h**	5th	38.67 ± 1.09 ^A^	42.33 ± 1.56 ^A^	36.67 ± 3.16 ^A^	40.50 ± 1.34 ^A^
	10th	46.50 ± 1.33 ^A^	49.67 ± 1.61 ^A^	61.50 ± 3.28 ^A^	66.33 ± 4.24 ^A^
**Red 23 h**	5th	41.67 ± 0.84 ^A^	41.67 ± 1.05 ^AB^	40.50 ± 1.12 ^A^	41.67 ± 2.12 ^A^
	10th	50.17 ± 0.83 ^A^	48.50 ± 1.45 ^A^	67.00 ± 2.80 ^A^	61.50 ± 3.91 ^A^
**Mix 17 h**	5th	42.17 ± 1.14 ^A^	42.67 ± 0.95 ^AB^	42.17 ± 2.84 ^A^	41.17 ± 0.95 ^A^
	10th	50.33 ± 1.26 ^A^	51.50 ± 1.11 ^A^	67.00 ± 3.09 ^A^	67.50 ± 4.03 ^A^
**Mix 23 h**	5th	39.67 ± 0.99 ^A^	38.00 ± 0.73 ^B^	39.67 ± 2.85 ^A^	36.67 ± 1.99 ^A^
	10th	47.33 ± 2.16 ^A^	50.00 ± 1.73 ^A^	62.33 ± 3.82 ^A^	62.50 ± 3.54 ^A^
**139 DIT**					
**Red 17 h**	5th	39.17 ± 1.19 ^A^	38.83 ± 0.91 ^A^	35.00 ± 2.41 ^A^	33.67 ± 1.87 ^A^
	10th	45.17 ± 0.83 ^A^	46.83 ± 1.19 ^A^	56.67 ± 1.86 ^A^	55.50 ± 2.49 ^A^
	15th	47.17 ± 2.09 ^A^	45.17 ± 1.01 ^A^	56.50 ± 3.66 ^A^	57.50 ± 3.02 ^A^
**Red 23 h**	5th	37.50 ± 0.92 ^A^	40.33 ± 1.02 ^A^	35.17 ± 3.17 ^A^	38.00 ± 2.28 ^A^
	10th	44.17 ± 2.47 ^A^	45.00 ± 1.75 ^A^	56.00 ± 2.77 ^A^	54.83 ± 2.41 ^A^
	15th	46.17 ± 1.22 ^A^	45.83 ± 1.38 ^A^	56.83 ± 2.56 ^A^	54.00 ± 1.29 ^A^
**Mix 17 h**	5th	37.67 ± 1.31 ^A^	39.33 ± 1.20 ^A^	34.50 ± 1.34 ^A^	35.17 ± 2.47 ^A^
	10th	42.17 ± 2.26 ^A^	43.33 ± 1.73 ^A^	53.83 ± 2.76 ^A^	49.67 ± 3.02 ^A^
	15th	44.33 ± 1.41 ^A^	49.17 ± 1.38 ^A^	50.83 ± 4.07 ^A^	55.50 ± 2.32 ^A^
**Mix 23 h**	5th	40.33 ± 1.54 ^A^	39.50 ± 0.89 ^A^	38.17 ± 1.42 ^A^	40.00 ± 1.73 ^A^
	10th	46.67 ± 1.76 ^A^	42.17 ± 1.19 ^A^	59.17 ± 2.47 ^A^	53.83 ± 2.50 ^A^
	15th	43.33 ± 2.30 ^A^	45.50 ± 1.71 ^A^	58.17 ± 2.89 ^A^	57.83 ± 2.23 ^A^

**Table 5:** Pigment analysis of plants grown under red 17 h, red 23 h, mix 17 h, and mix 23 h at 31 (16 December 2018), 60 (14 January 2019), and 139 DIT (3 April 2019). Values ± the standard error of the mean are representative of n = 6 for TE and TK plants under all lighting treatments. TE values are under white columns while TK values are under shaded columns. Different letter groups (A, B) represent a statistical difference between lighting treatments within a cultivar, time point, leaf rank, and pigment at *p* < 0.05.

		Chlorophyll *a* + *b* (µg cm^−2^)		Carotenoids (µg cm^−2^)	
Cultivar		TE	TK	TE	TK
**Lighting Treatment**	Leaf Rank				
**31 DIT**					
**Red 17 h**	5th	39.98 ± 0.90 ^A^	38.05 ± 2.79 ^A^	7.93 ± 0.15 ^A^	7.59 ± 0.47 ^A^
**Red 23 h**	5th	38.19 ± 1.68 ^A^	36.62 ± 1.21 ^A^	7.63 ± 0.28 ^A^	7.37 ± 0.20 ^A^
**Mix 17 h**	5th	38.61 ± 1.72 ^A^	38.80 ± 1.74 ^A^	7.70 ± 0.29 ^A^	7.73 ± 0.29 ^A^
**Mix 23 h**	5th	40.11 ± 1.81 ^A^	40.44 ± 1.13 ^A^	7.94 ± 0.39 ^A^	8.00 ± 0.19 ^A^
**60 DIT**					
**Red 17 h**	5th	26.77 ± 1.36 ^B^	31.47 ± 1.28 ^A^	5.68 ± 0.24 ^B^	6.50 ± 0.22 ^A^
	10th	34.30 ± 1.42 ^B^	36.61 ± 1.02 ^AB^	6.98 ± 0.24 ^B^	7.37 ± 0.29 ^A^
**Red 23 h**	5th	29.31 ± 2.31 ^B^	29.91 ± 1.91 ^AB^	6.21 ± 0.51 ^B^	6.22 ± 0.34 ^AB^
	10th	34.49 ± 2.17 ^B^	33.75 ± 0.96 ^AB^	7.00 ± 0.37 ^B^	6.89 ± 0.21 ^A^
**Mix 17 h**	5th	39.47 ± 3.49 ^A^	38.21 ± 2.16 ^A^	8.45 ± 0.77 ^A^	7.63 ± 0.36 ^A^
	10th	41.46 ± 2.02 ^A^	38.62 ± 1.36 ^A^	8.17 ± 0.33 ^A^	8.26 ± 0.39 ^A^
**Mix 23 h**	5th	28.47 ± 2.73 ^B^	22.75 ± 2.42 ^B^	5.98 ± 0.60 ^B^	4.95 ± 0.44 ^B^
	10th	34.63 ± 1.07 ^AB^	32.36 ± 1.34 ^B^	7.04 ± 0.18 ^B^	6.88 ± 0.38 ^A^
**139 DIT**					
**Red 17 h**	5th	40.94 ± 1.89 ^A^	41.94 ± 1.25 ^B^	8.08 ± 0.31 ^A^	8.25 ± 0.20 ^B^
	10th	43.86 ± 1.54 ^A^	44.16 ± 1.25 ^A^	8.56 ± 0.25 ^A^	8.61 ± 0.20 ^A^
	15th	34.10 ± 2.20 ^A^	37.82 ± 2.02 ^A^	6.94 ± 0.37 ^A^	7.57 ± 0.34 ^A^
**Red 23 h**	5th	42.59 ± 0.54 ^A^	42.78 ± 0.86 ^B^	8.36 ± 0.09 ^A^	8.39 ± 0.14 ^B^
	10th	44.80 ± 1.76 ^A^	45.43 ± 0.94 ^A^	8.71 ± 0.29 ^A^	8.82 ± 0.15 ^A^
	15th	37.11 ± 1.16 ^A^	32.57 ± 1.55 ^A^	7.45 ± 0.19 ^A^	6.68 ± 0.26 ^A^
**Mix 17 h**	5th	43.26 ± 1.17 ^A^	46.36 ± 1.13 ^A^	8.46 ± 0.19 ^A^	8.97 ± 0.18 ^A^
	10th	45.19 ± 2.26 ^A^	44.43 ± 1.09 ^A^	8.77 ± 0.36 ^A^	8.65 ± 0.18 ^A^
	15th	35.89 ± 3.57 ^A^	38.33 ± 1.94 ^A^	7.22 ± 0.61 ^A^	7.65 ± 0.32 ^A^
**Mix 23 h**	5th	45.65 ± 2.23 ^A^	44.30 ± 1.31 ^AB^	8.85 ± 0.36 ^A^	8.63 ± 0.21 ^AB^
	10th	42.53 ± 1.06 ^A^	45.38 ± 0.87 ^A^	8.35 ± 0.17 ^A^	8.81 ± 0.14 ^A^
	15th	34.38 ± 2.25 ^A^	31.98 ± 1.89 ^A^	6.98 ± 0.38 ^A^	6.58 ± 0.33 ^A^

**Table 6:** Monthly yield analysis for cv. ‘Trovanzo’ grafted onto cv. ‘Emperator’ (TE) or onto cv. ‘Kaiser’ (TK), grown under red 17 h, red 23 h, mix 17 h, and mix 23 h lighting treatments. Values represent the mean ± the standard error of the mean, where n = 4 for TE and TK. TE values are under white columns while TK values are under shaded columns. Of note, values representing May yield include a strip harvest on 22 May 2019. Total yield is the summation of the harvest period. Within each yield parameter, month (or total), and rootstock, letter groups (A, B, C) represent a statistical difference as determined by a two-way ANOVA with a Tukey–Kramer adjustment (*p* < 0.05). *p* values at the bottom of the table are representative of the cumulative data after a two-way ANOVA.

	Fruit per Stem		Fruit Weight per Stem (kg stem^−1^)	
Cultivar	TE	TK	TE	TK
**February**				
**Lighting Treatment**				
**Red 17 h**	9 ± 1 ^A^	8 ± 2 ^A^	0.68 ± 0.06 ^AB^	0.63 ± 0.07 ^A^
**Red 23 h**	7 ± 2 ^A^	10 ± 1 ^A^	0.52 ± 0.10 ^B^	0.67 ± 0.05 ^A^
**Mix 17 h**	11 ± 2 ^A^	8 ± 2 ^A^	0.88 ± 0.12 ^A^	0.65 ± 0.15 ^A^
**Mix 23 h**	7 ± 1 ^A^	8 ± 1 ^A^	0.50 ± 0.04 ^B^	0.52 ± 0.02 ^A^
**March**				
**Red 17 h**	21 ± 2 ^A^	22 ± 2 ^A^	1.66 ± 0.20 ^AB^	1.90 ± 0.14 ^A^
**Red 23 h**	16 ± 2 ^A^	17 ± 1 ^BC^	1.13 ± 0.16 ^AB^	1.07 ± 0.08 ^B^
**Mix 17 h**	20 ± 2 ^A^	21 ± 2 ^AB^	1.70 ± 0.10 ^A^	1.72 ± 0.12 ^A^
**Mix 23 h**	17 ± 2 ^A^	12 ± 1 ^C^	0.97 ± 0.11 ^B^	0.70 ± 0.03 ^C^
**April**				
**Red 17 h**	20 ± 1 ^A^	22 ± 1 ^A^	1.97 ± 0.26 ^A^	2.23 ± 0.13 ^A^
**Red 23 h**	22 ± 2 ^A^	21 ± 1 ^A^	2.06 ± 0.14 ^A^	1.77 ± 0.07 ^B^
**Mix 17 h**	19 ± 1 ^A^	22 ± 1 ^A^	1.93 ± 0.15 ^A^	2.05 ± 0.10 ^AB^
**Mix 23 h**	16 ± 1 ^B^	12 ± 1 ^B^	1.42 ± 0.06 ^A^	0.95 ± 0.05 ^C^
**May**				
**Red 17 h**	26 ± 1 ^A^	24 ± 1 ^A^	3.47 ± 0.09 ^A^	3.32 ± 0.17 ^A^
**Red 23 h**	24 ± 1 ^A^	25 ± 2 ^A^	3.50 ± 0.10 ^A^	3.22 ± 0.10 ^A^
**Mix 17 h**	25 ± 1 ^A^	27 ± 1 ^A^	3.47 ± 0.10 ^A^	3.45 ± 0.13 ^A^
**Mix 23 h**	25 ± 1 ^A^	26 ± 1 ^A^	3.66 ± 0.14 ^A^	3.37 ± 0.21 ^A^
**Cumulative**				
**Red 17 h**	74 ± 1 ^A^	75 ± 3 ^A^	7.29 ± 0.17 ^AB^	8.08 ± 0.36 ^A^
**Red 23 h**	72 ± 1 ^A^	72 ± 1 ^A^	7.51 ± 0.08 ^A^	6.75 ± 0.11 ^B^
**Mix 17 h**	73 ± 1 ^A^	77 ± 2 ^A^	7.73 ± 0.15 ^A^	7.87 ± 0.20 ^A^
**Mix 23 h**	64 ± 1 ^B^	57 ± 2 ^B^	6.54 ± 0.16 ^B^	5.54 ± 0.20 ^C^

## Data Availability

All data reported can be found within the manuscript.

## Abbreviations

CAB-13: type III light harvesting chlorophyll a/b binding protein 13; CL: Continuous lighting; DIT: Days into the treatment; DLI: Daily light integral; F_o_: Minimum fluorescence in a dark-adapted state; F_m_: Maximum fluorescence in a dark-adapted state; F_v_: Variable fluorescence in a dark-adapted state; F_v_/F_m_: Maximum quantum efficiency of PSII; J_max_: Maximum rate of electron transport; LCP: Light compensation point; LED: Light-emitting diode; LUE: Light use efficiency; N: Photon flux; NCER: Net carbon exchange rate; PHY A: Phytochrome A; Pn_max_: Photosynthetic maximum; PPE: Photosynthetic photon efficacy; PSI: Photosystem I; PSII: Photosystem II; PSS: Phytochrome photostationary state; QY; Quantum yield; R:Fr: Red to far-red ratio; TE: ‘Trovanzo’ grafted onto ‘Emperator’; TK: ‘Trovanzo’ grafted onto ‘Kaiser’; V_cmax_: Maximum rate of Rubisco carboxylation; WUE: Water use efficiency; σ_r_: Photochemical cross-section of phytochrome in the red absorbing state; σ_F__r_: Photochemical cross-section of phytochrome in the red absorbing state.
